# Regression of Neonatal Cardiac Rhabdomyoma in Two Months Through Low-Dose Everolimus Therapy: A Report of Three Cases

**DOI:** 10.1007/s00246-017-1688-4

**Published:** 2017-08-05

**Authors:** Jeng-Sheng Chang, Ping-Yun Chiou, Shu-Hui Yao, I-Ching Chou, Ching-Yuang Lin

**Affiliations:** 1 Division of Pediatric Cardiology, China Medical University Children’s Hospital, 2 Yu-Der Rd., North district, Taichung, 40447 Taiwan; 2 Pediatric Neurology, China Medical University Children’s Hospital, Taichung, 40447 Taiwan; 3 Pediatric Nephrology, China Medical University Children’s Hospital, Taichung, 40447 Taiwan; 4 Department of Pharmacy, China Medical University Hospital, Taichung, 40447 Taiwan; 5 School of Medicine, College of Medicine, China Medical University, Taichung, 40447 Taiwan; 6 Graduate Institute of Integrated Medicine, College of Chinese Medicine, China Medical University, Taichung, 40447 Taiwan

**Keywords:** Cardiac rhabdomyoma, Everolimus, Neonate, Side effect

## Abstract

Cardiac rhabdomyoma (CR) is the most common cardiac tumor in newborns. Approximately 75% of cases are associated with tuberous sclerosis complex. Although these tumors usually spontaneously regress after 2 years of age, they can be life-threatening when they obstruct major cardiac inflow or outflow pathways. Everolimus is an inhibitor of the mammalian target of rapamycin, reducing its production of the proteins harmartin and tuberin. Everolimus has demonstrated a remarkable suppression effect in children with tuberous sclerosis complex at doses of 4.7–5.6 mg/M^2^/day and serum trough levels of 5–15 ng/mL. Since 2012, five case reports of neonates with CR have also reported the tumor-regressing effect of everolimus. However, the optimal dosage for neonates is still unknown. Over the past 2 years, we have deliberately used a low dose everolimus regimen (0.3–0.67 mg/M^2^/day) in three neonates with large CRs, in an effort to maintain serum trough levels at 3–7 ng/mL. In all three cases, the tumors regressed smoothly within 2 months. Regarding the drug’s side effect of predisposing patients to infection, we observed that adenovirus pneumonia occurred in one case at 3 months of age, and chicken pox occurred in another case at 9 months of age; both recovered smoothly. Our three cases of neonatal CR demonstrate that a low-dose everolimus regimen is an effective treatment for tumor regression.

## Background

Cardiac tumors occur in only 0.027% of children, and cardiac rhabdomyoma (CR) comprises 45% of these occurrences [1]. Approximately 70–90% of CR patients also present with cerebral tuberous sclerosis, exhibiting a phenotype of tuberous sclerosis complex (TSC) [2]. Pathologically, CR is a type of hamartoma composed of striated muscles that usually follows a benign course of spontaneous regression after 2 years [1, 3]. However, when a CR severely obstructs the cardiac inflow or outflow tract of a neonate, urgent cardiac surgery to remove the tumors must be considered [1–5]. Since 2012, five case reports have been published in the relevant English literature that present successful cases of using everolimus, an inhibitor of the mammalian target of rapamycin (mTOR), to trigger tumor regression in neonatal patients with CR [6–10]. These case reports offer hope of nonsurgical survival among high-risk patients. However, the proper dosage and duration of using everolimus in neonates, and knowledge about avoiding its side effects are largely unknown [2]. Over the past 2 years, we have observed three neonates prenatally diagnosed with CR, including one with total left ventricular obstruction. Attempting to reduce the drug’s potential side effects as much as possible, we used a lower-than-recommended-dosage regimen of everolimus on all three patients.

## Case 1

**Fig. 1:**
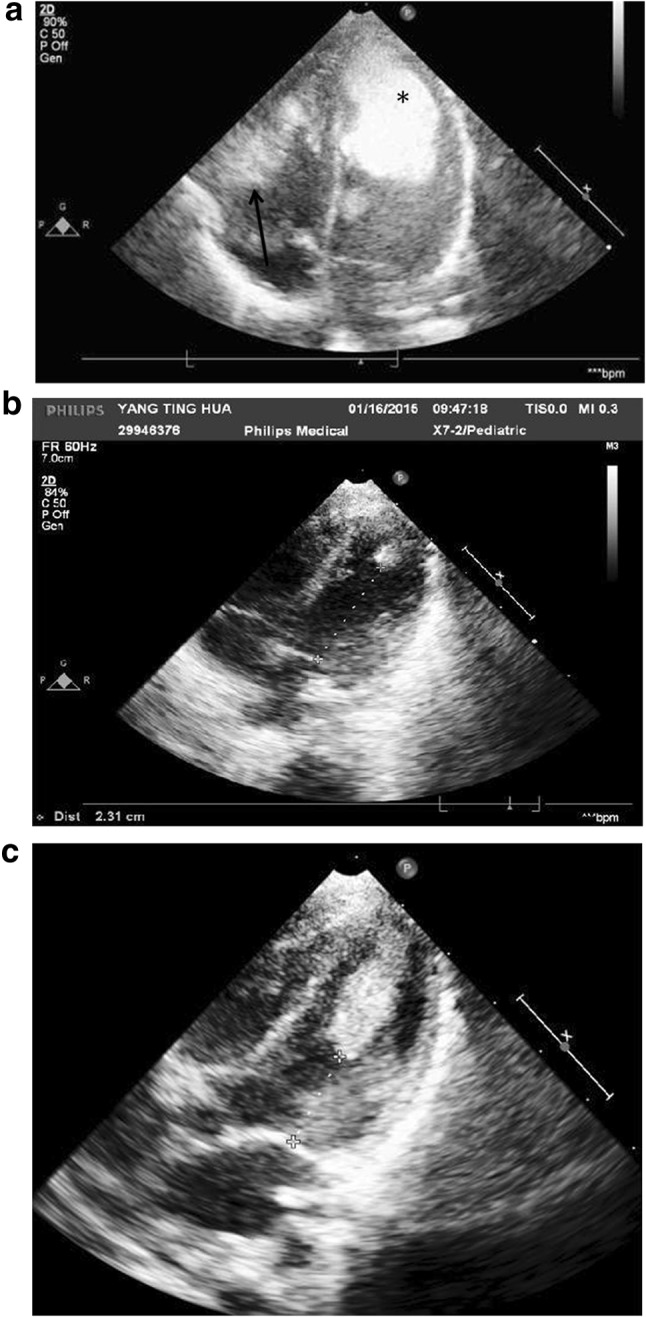
Case 1: **a** Echocardiogram of the first day of life. The LV tumor *Asterisk* occupied its apical half. The *arrow* indicates another small tumor in the RV free wall. **b** After 3 months of everolimus therapy, the LV tumor has regressed to a small nodule in the apex (*arrow*). **c** Two months after everolimus cessation, the tumor significantly rebounded; however, the LV chamber has grown to a satisfactory size

A 38-week-old female neonate, born to a 35-year-old woman by cesarean section, was found to have a large, high echogenic cardiac tumor occupying the apical half chamber of the left ventricle (LV), and a small tumor in the right ventricle (RV) free wall, suggesting a case of CR. The baby was born weighing 2800 gm, with pink skin color, cool limbs, tachycardia (150 bpm), tachypnea (40 per min’), and subcostal retractions. Electrocardiography showed a normal sinus rhythm, and echocardiographic measurements of the LV tumor by 4-chamber view revealed a surface area of 2.19 cm^2^ (Fig. 1a). Although the LV ejection fraction in the parasternal long axis view remained normal (69.2%), its compliance appeared decreased, as reflected by a reversed E/A ratio in the mitral valve flows (0.46/0.66 M/s), a high Tei index (0.9), and a low *Z* score (0.75). Additionally, the brain and renal ultrasound studies were both normal.

After providing an explanation to the parents, we began an off-label use of oral low-dose everolimus therapy (0.0625 mg/day, 1/4 of a 0.25 mg tablet, equivalent to 0.334 mg/M^2^/day), aiming to achieve a trough serum everolimus level of 3–7 ng/mL. Her cardiac function was also supported with digoxin and furosemide. We monitored the complete blood counts, renal and liver functions, and serum everolimus levels once a week. After the first week, the everolimus level was 3.50 ng/mL. We escalated the dosage to 1/3 tablet a day (0.427 mg/M^2^/day) for the second week, and 1/2 tablet a day (0.558 mg/M^2^/day) for the third week. Echocardiography revealed a remarkable reduction of tumor size. With the same dose, she was discharged on the 17th day of therapy. By the 50th day of therapy, the LV tumor had reduced to 0.29 cm^2^ surface area, approximately one-eighth of the original size (Fig. 1b). However, the everolimus serum level unexpectedly rose to 20 ng/mL (Fig. 4) and we tapered the dosage back to 1/4 tablet a day (0.241 mg/M^2^/day). The serum level remained high (14.18 ng/mL) on the 80th day of therapy, so we further decreased the dosage to 1/8 tablet a day (0.12 mg/M^2^/day). Two weeks later, she suffered fever, cough and respiratory distress. Everolimus was withheld by the parents, and she was readmitted to our ward. Her weight (3.9 kg), height (55 cm) and head girth (39 cm) were all below the third percentile for an average 3-month-old female infant. The chest roentgenogram showed an interstitial type of pneumonia, and adenovirus was isolated from her throat. Because the everolimus serum level had become immeasurable (<1.5 ng/mL), we suspended the therapy. She was discharged after 1 week. Later, follow-up echocardiograms noticed a slow but steady increase in the tumor size, whose surface areas were 0.69 cm^2^ at 4 months, 0.86 cm^2^ at 5 months, 1.05 cm^2^ at 9 months, and 1.13 cm^2^ at 1-year-and-4 months of age (Fig. 1c). However, with growing of the LV chamber and its improving compliance, the cardiac output remained adequate. By the age of 1-year-11-months, her bodily statistics had improved to the 50–70th percentile for her age: she weighed 10 kg, was 93.8 cm tall, and had a head circumference of 47.5 cm. Finally, the genetic studies for TSC-1 and TSC-2 anomalies were both negative.

## Case 2

**Fig. 2:**
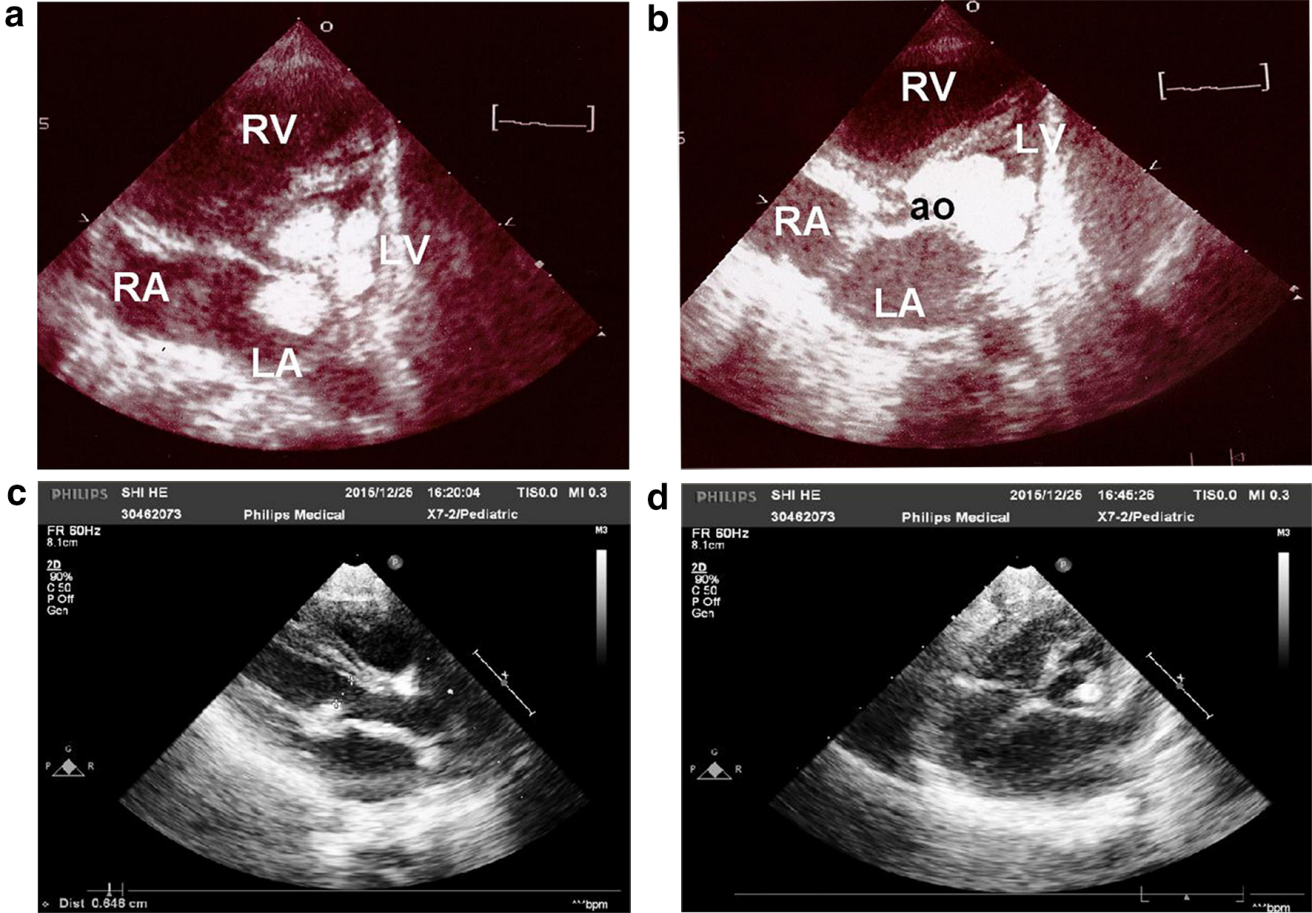
Case 2: **a**, **b** Echocardiogram on the second day of life, showing a 3-lobe LV tumor completely obstructed the mitral valve orifice and the subaortic area. **c**, **d** After 2 months of everolimus therapy, the LV tumor regressed to a small nodule attached at the lateral papillary muscle of the mitral valve. Both the mitral orifice and the subaortic area now have adequate room for blood flow

A 37-week-old, 2880 gm male neonate, born to a G6P3A3, 40-year-old mother, was transferred to the pediatric intensive care unit of this tertiary referral hospital from an obstetric hospital on his second day of life because of a large cardiac tumor that had completely obstructed the inflow and outflow tracts of the LV. The cardiac tumor had been observed by fetal ultrasonography since the 32nd week of pregnancy, and was assumed to be a nonsurviving case. Upon admission, the patient was in distress, with tachypnea (60 per min), tachycardia (160 beats per min), grunting sounds, and hypoxemia (SaO2 85%). He immediately received tracheal intubation and a dopamine infusion. Echocardiography revealed a large, high echogenic tumor with three lobes in the basal area of the LV. Its wide base was attached firmly at the outlet septum, extended to the LV free wall, and involved the lateral papillary muscle of the mitral valve. The LV inflow, the mitral valve orifice, and the LV outflow (i.e., the subaortic area) were completely occluded by the tumor (Fig. 2a, b). Hence, all of the pulmonary venous return flows had to be shunted across the atrial septal defect, mixed with the systemic venous returns in the right heart, and pumped together into the main pulmonary artery (MPA). The MPA flow then divided a large portion of its flow across the ductus arteriosus, providing complete aortic blood flow. Thus, the patient was dependent on single ventricle circulation to survive.

Considering its difficulty and high risk of mortality, our cardiac surgeon elected to not perform tumor resection. After a consultation with the parents, they agreed to off-label everolimus therapy. We began the therapy at 0.0625 mg/day (1/4 tablet, 0.316 mg/M^2^/day), in addition to a combination of intravenous vasoactive drugs (namely, dopamine, bosmin, and prostaglandin E1). Although cerebral ultrasound did not find a significant tubercle in the brain, a generalized seizure occurred the next day, so the anticonvulsant therapy of dilantin and phenobarbital was begun. The everolimus serum level was 5.53 ng/mL on the 7th day of therapy, but decreased to 1.68 ng/mL on the 14th day, which was assumed to be caused by a drug–drug interaction with anticonvulsants (Fig. 4). Nevertheless, echocardiography observed an initiation of tumor regression, and both mitral flow and subaortic flow along the shrunken tumor appeared. We then increased the everolimus dosage to 0.125 mg/day (0.613 mg/M^2^/day), but the serum level remained at 1.92 ng/mL on the 21st day, and even dropped to less than 1.5 ng/mL on the 28th day, when the phenobarbital serum level rose to 80 mg/L. Thereafter, we reduced the phenobarbital dosage and increased everolimus to 0.25 mg/day (1.2 mg/M^2^/day). On the 35th day, the serum level rose to 5.59 ng/mL (Fig. 4). Another echocardiogram displayed a remarkable regression of the tumor, and blood flow through both the mitral and aortic valves became satisfactory. We ended all the courses of anticonvulsants and reduced everolimus to 0.125 mg/day (0.6 mg/M^2^/day). Finally, the endotracheal tube was removed on the 37th day of therapy. The patient was discharged on the 74th day of therapy when his weight was 4.4 kg (3rd percentile) and his height was 56 cm (5–15th percentile).

After discharge, the tumor continued to reduce in size. At 6 months of age, under an everolimus dose of 0.35 mg/M^2^/day (serum level: 1.81 ng/mL), the remaining tumor appeared as only a small nodule attached at the lateral papillary muscle of the LV (Fig. 2c, d). We thus suspended everolimus therapy. However, 16 days later, the shrunken tumor rebounded to a surface area of 1.35 cm^2^ producing a mild degree of mitral regurgitation. Everolimus therapy was reinstituted at 0.125 mg/day (1/4 tablet bid, 0.35 mg/M^2^/day). Although all serum levels afterwards were less than 1.5 ng/mL, the tumor remained as a small nodule throughout follow-up. At 1 year of age, his weight (8.8 kg), height (75 cm) and head girth (45.5 cm) had all improved to the 15–50th percentile for his age, his development was normal, and the gene studies of TSC-1 and TSC-2 were both negative.

## Case 3

**Fig. 3:**
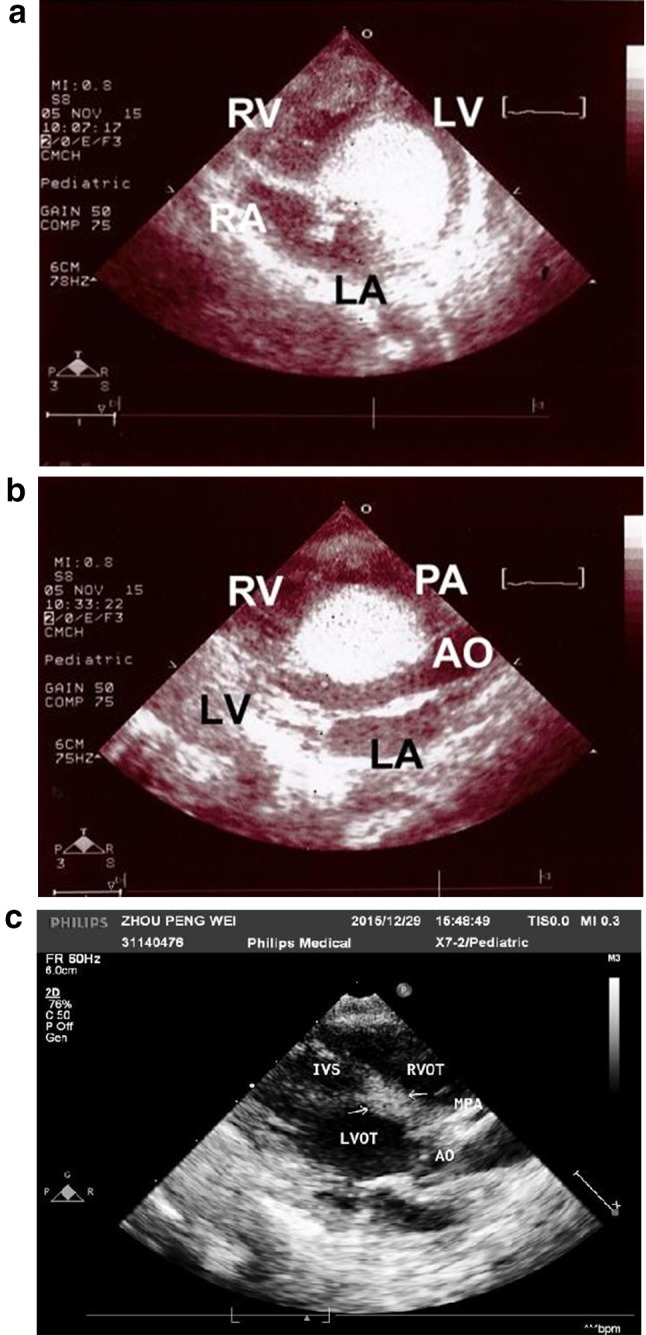
Case 3: **a**, **b** Echocardiogram on first day of life. **c** Echocardiogram after 2 months of everolimus therapy

**Fig. 4:**
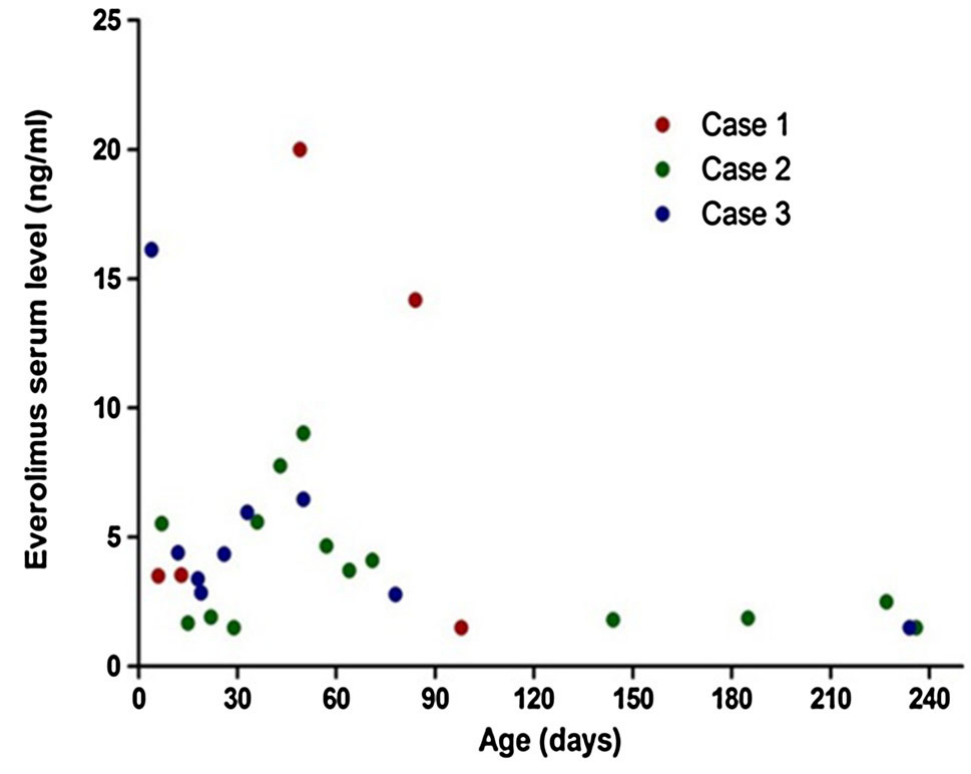
Distribution of everolimus trough serum levels of the three patients. With dosage ranging between 0.3 and 0.67 mg/M^2^/day, the average serum level was 5.14 ± 4.62 ng/mL (4.96 ± 3.61 ng/mL during hospitalizations of 17, 74, and 32 days, respectively). Aside from the three unusually high levels, 92% of the serum samples were <7.0 ng/mL

A fetal ultrasound screening on a 35-year-old pregnant woman revealed a large (3.10 cm^2^), high echogenic tumor in the LV outlet septum of a 34-week-old male fetus. He was born by cesarean section at 37.5 weeks, with a weight (2.72 kg), height (48 cm), and head girth (33.5 cm) that were all within the 15–50th percentile. Echocardiography showed that the tumor not only exerted compression on the LV outflow tract, but also extended outward to the septum between the ascending aorta and the MPA, pushing them apart (Fig. 3a, b). The surface areas of the tumor were 2.60 cm^2^ in the long axis view and 4.10 cm^2^ in the short axis view. Electrocardiograms also detected frequent episodes of isolated ventricle premature contractions (VPC). We began everolimus therapy at 0.125 mg/day (0.658 mg/M^2^/day), along with a low dose of digoxin (5 μg/kg/day) on his second day of life. On the fifth day of therapy, the serum level was 16.12 ng/mL (higher than our target range); therefore, we reduced the daily dose to 0.0625 mg (0.33 mg/M^2^/day) (Fig. 4). The following three weekly serum levels of everolimus were 4.41, 3.39, and 2.86 ng/mL, respectively. When he was discharged on his 32nd day of life, we increased the dose to 0.125 mg/day (0.51 mg/M^2^/day). Three months later, echocardiography revealed a remarkable shrinkage of the tumor (0.80 cm^2^ in long axis view, Fig. 3c), but his weight and height had decreased to less than 15th percentile for age; thus, we suspended the everolimus therapy. However, follow-up echocardiograms revealed a steady regrowth of the tumor. Specifically, its surface area increased to 0.99 cm^2^ after 2 weeks and to 2.97 cm^2^ after 2 months; the compression effect on the LV outflow tract also recurred. When he was 5 months old, we reinstituted everolimus therapy at 0.125 mg/day (0.379 mg/M^2^/day). Although all of the follow-up serum levels were less than 1.5 ng/mL, the tumor regressed smoothly. At 9-months-old, he contracted chicken pox, and the therapy was stopped again. He recovered smoothly without using antichicken pox medication. At 10 months of age, his weight (8.3 kg) and head girth (44.5 cm) had improved to the 15–25th percentile for his age, but his height (70.5 cm) remained below the 15th percentile. The specific loci for TSC-1 and TSC-2 gene abnormality were also negative.

## Discussion

In this cases series of three neonates with CR, we deliberately used a low dose regimen of everolimus therapy, beginning with 0.3–0.67 mg/M^2^/day to facilitate a serum level between 3 and 7 ng/mL. All cardiac tumors regressed smoothly within 2 months. This dosage is considerably lower than the recommended dose of 4.5 mg/M^2^/day for managing cerebral subependymal giant cell astrocytomas (SEGA) in TSC children [10], which aims for a serum level of 5–15 ng/mL and has been demonstrated to be effective at reducing the tumor size of SEGAs by half in 35% of patients (*p* < 0.0001).

Although both SEGA and CR are hamartomas and should be responsive to everolimus therapy, we were unsure whether the recommended dosage and range of serum levels for 1–23.5 year-old children with SEGAs would be safe for neonatal patients with CR [1, 2, 10]. Therefore, we deliberately used a low-dose regimen to treat these patients. The distribution of the everolimus serum levels in all three patients indicated that most levels were under 7 ng/mL. Nevertheless, this low level was still effective at reducing the tumors in all three neonatal patients within 2 months (Fig. 4).

Since 2012, five case reports have described the effectiveness of using everolimus to treat neonatal patients with CR and substantial intracardiac obstructions [6–9, 11]. The idea of using everolimus to treat CR was derived from the successful experiences of using everolimus on patients with SEGA [2, 10]. Accordingly, the high-dose regimens for achieving serum trough levels between 5 and 15 ng/mL that had been applied to patients with SEGA, were used in the initial case reports [6, 7, 11]. The case report by Demir et al. (2012) was the pioneer instance of using everolimus to treat neonatal patients with CR. Initially, they used a high dose regimen of 0.25 mg every 6 h for four doses (equivalent to 5 mg/M^2^/day), which resulted in a high serum level of 83 ng/mL. That dosage was immediately reduced to 0.25 mg every 12 h, 2 days per week, and the tumor uneventfully regressed to a small size after 75 days of therapy [6]. Hoshal et al. used a high dose of everolimus (0.5 mg/day, equivalent to 2.5 mg/M^2^/day) on a 2-day-old neonate after a successful cardiopulmonary resuscitation. Although the tumor had obstructed the outflow tract of the RV and infiltrated into the LV myocardium, it was reduced to a small size within 2 months of therapy [7].

By contrast, we targeted a lower serum trough level (3–7 ng/mL) from the very beginning in all three of the cases presented herein by using a lower dosage (0.3–0.67 mg/M^2^/day). Notably, the tumors in all three of our patients successfully regressed in 2 months. Despite our efforts to maintain a low-dose everolimus regimen throughout, two occasions of unexpectedly high serum levels still occurred. One high serum level (16.1 ng/mL) occurred in a 4-day-old neonate who was receiving a dose of 0.67 mg/M^2^/day. Another high level (over 20 ng/mL) occurred in a 49-day-old infant who was receiving a dose of 0.56 mg/M^2^/day at home; she also suffered adenovirus pneumonia immediately following this high-serum-level episode. These two occasions of high serum levels reflect the unpredictable nature of drug metabolism during early infancy, and suggest the impairment of hepatic drug metabolism following a viral infection [12].

Maintaining an adequate serum level during acute CR is difficult if the patients are also receiving drugs that interact with everolimus. For example, we used luminal and dilantin to control seizure attacks in our Case 2 patient. These two anticonvulsants are *CYP3A4/P*- *glycoprotein(PgP) Inducers* and possess a drug–drug interaction with everolimus [12]. Accordingly, the serum levels of everolimus dropped from 5.53 ng/mL to an undetectable level. We subsequently had to escalate the patient’s everolimus dosage from 0.3 to 0.6 to 1.2 mg/M^2^/day, until his serum levels returned to 5.59 ng/mL.

Two cases of using low-dose everolimus to manage neonatal CR from its outset have recently been reported. First, Goyer used a dose of 0.65 mg/M^2^/day in two premature neonatal patients. Their cardiac tumors, which exhibited significant hemodynamic derangement, regressed smoothly, and the drugs were discontinued on day 36 for one patient and on day 81 for the other [8]. Bornaun also used a low-dose everolimus course of 0.25 mg two times a day, and twice a week (equivalent to 0.7 mg/M^2^/day), and aimed for a serum trough level of 2.6–6.1 ng/mL. Although the actual serum levels of that patient were only between 0.4 and 2.6 ng/mL, Bornaun did not try to increase the dose, and the tumor regressed in 4 weeks [9]. Therefore, including our case series, a total of six case reports have demonstrated that a dose of everolimus of less than 0.7 mg/M^2^/day is effective for producing regression of neonatal CR.

Notably, everolimus is an immunosuppressive drug that may predispose recipients to a higher risk of infection, particularly by opportunistic pathogens [12]. In our case series, for example, two episodes of unexpected infection occurred. Case 1 contracted adenovirus bronchopneumonia when she was 3 months of age, which occurred immediately following unexpectedly high drug levels (20 ng/mL). Case 3 contracted chicken pox when he was 9 months of age, though the drug dosage had already been tapered to 0.33 mg/M^2^/day and the serum levels were less than 1.5 ng/mL. Although both patients smoothly recovered from these infection episodes, they demonstrated the threatening infections that can be triggered in everolimus-treated infants.

Everolimus can also inhibit the formation of the human protein mTOR, which suggests that the drug may inhibit children’s growth [12]. In all three of our cases, we found that growth was stunted during their early infancy. For example, at 3 months of age, the patients’ body weights dropped to the 3rd, 3rd, and 15th percentile for their ages, respectively. Later, as we tapered the doses, their body weights all improved to 15–50th percentile. Ideally, everolimus would be administered to neonatal patients using the minimum possible dosage and duration. We tried to suspend the drugs when our patients were 3–6 months old, but found that the tumors rebounded in 2–4 weeks (Fig. 1c). We readministered everolimus to our Case 2 patient because of the tumor-related mitral regurgitation, and to our Case 3 patient because of the compression on the left ventricle outflow tract.

Although surgical resection of the obstructive CR through open heart surgery is a considerably more invasive procedure than our using tumor suppression medication [5–7, 13], surgery might still play a critical role in some cases. For example, we treated one neonatal patient with CR three years ago who had a tumor growing from the free wall of the RV outflow tract, which was severely obstructing outflow in the RV. We successfully applied tumor resection (not reported in this paper). Therefore, because of the tumor-specific characteristics of different neonates, treatment plans should always be individualized.

Everolimus therapy must be administered for at least 2 weeks before a significant reduction in tumor size can occur. Therefore, during that period of hemodynamic instability, the presence of a highly integrated cardiac intensive care team is crucial. In Hoshal’s case, they had to perform cardiopulmonary resuscitation before any medication could begin [7]. In Demir’s and Bornaun’s cases, and our Case 2 patient, continuous prostaglandin infusions had to be used to maintain the patient’s ductal arteriosus sufficiently open. Moreover, multiple cardiovascular drugs including dopamine, milrinone, and bosmin were provided [6, 11]. Therapeutic cardiac catheterization procedures, including PDA stent and balloon atrioseptostomy, were undertaken in Mlczoch’s case [11]. Similarly, because the CR had totally obstructed the mitral and aortic areas in our case 2 patient, he was dependent on ventilator support through tracheal intubation until the tumor resolved (Fig. 2a–d).

Some proposed natural timings of spontaneous CR regression showed a wide range of variation (range 0–105 months, median 4.3 months) [3]. In our 3-cases series, we have observed that significant tumor rebounds occurred in two patients, two to four weeks after suspension of everolimus therapy at 3 months and 6 months of age, respectively. Because most CR may regress to only small tumors within the first year of life, it is never necessary to treat an asymptomatic CR patient. However, for the symptomatic cases of neonatal CR, including heart failure, arrhythmia or cyanosis, the patients will need specific medications and close observation in hospital until their symptoms end. When CR causes severe obstructions on the heart of a neonate, as that of our case 2, a life-threatening situation emerged. Before the use of everolimus therapy was advocated, most reported cases of obstructive CR had received tumor resections [1–4, 13]. Only one case report with severe obstruction found spontaneous tumor regression in 3 weeks without surgery [3].

In summary, a low-dose everolimus treatment course, in the range of 0.3–0.67 mg/M^2^/day that aimed for serum levels between 3 and 7 ng/mL, appeared effective at triggering tumor regression in neonatal patients with CR within 2 months. However, its potential predisposition of patients to infections and its inhibiting growth should be cautiously attended to.
